# Repurposing Clozapine: An Off-Label Approach to Target Impulsivity, Emotional Dysregulation, and Suicidality in Borderline Personality Disorder

**DOI:** 10.1192/j.eurpsy.2025.550

**Published:** 2025-08-26

**Authors:** B. Firenzuoli, A. Cuomo, E. Mariantoni, A. Morrone, P. Carmellini, S. Pardossi, A. Pierotti, A. Fagiolini

**Affiliations:** 1 Molecular Medicine, University of Siena, Siena, Italy

## Abstract

**Introduction:**

Clozapine, traditionally prescribed for treatment-resistant schizophrenia, has shown potential for off-label use, particularly in affective and personality disorders characterized by severe impulsivity, nonsuicidal self-injury (NSSI), and suicidality (Delgado *et al* 2020*, Journal of psychiatric research, 125*, 21-27). Emerging evidence suggests that clozapine’s unique pharmacodynamic profile (serotonergic activity and high affinity for D4R, α1R, H1R) may contribute to reducing aggression and impulsivity and offer therapeutic benefits for borderline personality disorder (BPD). This is particularly relevant given the high suicide risk in BPD patients, with an estimated annual rate up to 10%, 50 times higher than the general population. Despite this, current treatment guidelines for BPD typically limit pharmacological interventions in favor of psychotherapeutic approaches (Pascual JC *et al* Int Clin Psychopharmacol. 2010, 25(6), 349-55). However, clozapine’s ability to modulate impulsivity and emotional dysregulation could provide a valuable adjunct in the treatment of this complex disorder.

**Objectives:**

To evaluate the efficacy of clozapine in reducing impulsivity, emotional dysregulation, and suicidality in patients with BPD.

**Methods:**

The study cohort consisted of 47 patients (29 women) with a mean age of 28 years (IQR: 22-44), all diagnosed with BPD resistant to previous pharmacological treatments. Clozapine was introduced after a comprehensive risk-benefit assessment. At baseline, 96% of patients were taking mood stabilizers or anticonvulsants, 87% were taking SSRI/SNRI antidepressants, 81% were taking antipsychotics, and 66% were taking other medications such as benzodiazepines or gabapentinoids. Clinical assessments using the RIPoST-40, MOAS, and Columbia scales were administered at baseline, one week (T1), one month (T2), and three months (T3). Data were analyzed using repeated measures ANOVA and Friedman’s test with significance set at p<0.01 after Bonferroni correction.

**Results:**

Significant reductions in all scores were observed at T3: RIPoST-40 scores decreased by 40.15% (p<0.0001), MOAS by 58%(p<0.0001), Columbia Scale scores by 70.20% (p<0.0001). At month 3, clinical response, defined as a ≥50% reduction in scores, was achieved by 11% of patients on the RIPoST-40, 70% on the MOAS, and 94% on the Columbia Scale.

**Conclusions:**

Clozapine demonstrated significant reductions in impulsivity, emotional dysregulation, and suicidality in patients with BPD, with rapid improvements observed within the first week and sustained through 3 months. These findings suggest that clozapine, in combination with psychotherapy, may be an effective treatment strategy for the most severe symptoms of BPD. Further prospective studies with larger cohorts are needed to validate these preliminary results and to assess the long-term safety and efficacy of clozapine in this population.

**Disclosure of Interest:**

None Declared

